# Relationships of Body Composition with Refractive Error and Axial Length in 8-Year-Old Japanese Children

**DOI:** 10.3390/jcm15176883

**Published:** 2026-09-05

**Authors:** Mingxue Bao, Ryo Harada, Natsuki Okabe, Yuka Kasai, Airi Takahashi, Chio Kuleshov, Yumi Shigemoto, Ryoji Shinohara, Hideki Yui, Anna Kobayashi, Megumi Kushima, Sanae Otawa, Zentaro Yamagata, Kenji Kashiwagi

**Affiliations:** 1 Department of Ophthalmology, School of Medicine, University of Yamanashi, Chuo 409-3898, Japan; g23ddm19@yamanashi.ac.jp (M.B.); haradryo@gmail.com (R.H.); nokabe@yamanashi.ac.jp (N.O.); kasayuka@yamanashi.ac.jp (Y.K.); iairi@yamanashi.ac.jp (A.T.); chiok@yamanashi.ac.jp (C.K.); yumishig@yamanashi.ac.jp (Y.S.); 2 Center for Birth Cohort Studies, Interdisciplinary Graduate School of Medicine, University of Yamanashi, Chuo 409-3898, Japan; rshinohara@yamanashi.ac.jp (R.S.); hyui@yamanashi.ac.jp (H.Y.); anna_vfk@yahoo.co.jp (A.K.); kumegumi@yamanashi.ac.jp (M.K.); osanae@yamanashi.ac.jp (S.O.); zenymgt@yamanashi.ac.jp (Z.Y.)

**Keywords:** myopia, axial length, refractive error, Rohrer index, obesity, body composition, children, Japan Environment and Children’s Study

## Abstract

**Objectives:** This study aimed to examine the relationships between refractive error and axial length (AL) and body composition parameters, including obesity-related indices, in 8-year-old children enrolled in the Japan Environment and Children’s Study (JECS). **Methods:** From 2019 to 2022, data from the right eyes of 1866 children aged 8 years enrolled in the JECS adjunct study were analyzed. AL, noncycloplegic spherical equivalent (SE), and uncorrected visual acuity were measured. Relationships of these ocular parameters with height, weight, muscle mass, body fat percentage, the Rohrer index, and sex were evaluated. **Results:** The mean AL was 23.09 mm, and the mean SE was −0.52 D in the right eye. Despite the correlations of the Rohrer index with SE (r = 0.05, *p* = 0.02), uncorrected visual acuity expressed as logMAR (r = −0.07, *p* = 0.005) and AL (r = −0.06, *p* = 0.02) reached nominal statistical significance, and all correlation coefficients were close to zero, indicating no meaningful linear correlations. SE and logMAR showed no meaningful correlations with height, weight, muscle mass, or body fat percentage. AL showed small positive correlations with height (r = 0.17, *p* < 0.001) and muscle mass (r = 0.14, *p* < 0.001); its correlation with weight was negligible (r = 0.07, *p* = 0.004), and no meaningful correlation was observed with body fat percentage. **Conclusions:** Among 8-year-old children, the Rohrer index showed no meaningful linear correlation with refractive error, uncorrected visual acuity, or AL despite having nominally significant *p*-values. AL showed small positive correlations with height and muscle mass, suggesting a limited cross-sectional relationship with overall somatic size; however, the small effect sizes preclude strong conclusions. Because refraction was measured without cycloplegia, the SE findings should be interpreted cautiously, as accommodation may have shifted measurements in the myopic direction.

## 1. Introduction

The prevalence of myopia in children has increased rapidly in recent years [1], raising concerns about a future increase in myopia-related ocular diseases [2,3,4]. Although genetic factors contribute to myopia susceptibility [5], epidemiological studies have demonstrated that environmental factors and lifestyle habits also contribute to the onset and progression of myopia [6]. Consequently, various strategies and interventions have been developed to prevent or slow myopia progression [3]. Obesity has also been associated with a broad range of ocular diseases. Previous research has demonstrated that greater obesity is associated with an increased risk of cataract development [7] and elevated intraocular pressure [8], as well as a higher risk of age-related macular degeneration [8,9,10]. However, the relationship of obesity with refractive development in children remains incompletely understood. The Japan Environment and Children’s Study (JECS) is a nationwide prospective birth cohort study launched by the Japanese Ministry of the Environment in 2011, involving approximately 100,000 children with follow-up from birth onward. The primary objective of JECS is to identify environmental factors that influence children’s health and development and to contribute to creating a safe and healthy environment for child development. The Yamanashi Adjunct Study of the Japan Environment and Children’s Study (JECS-Y) is a multidisciplinary adjunct study of JECS involving approximately 4000 participants from Yamanashi Prefecture. As part of the ophthalmologic component of JECS-Y, we conducted a cross-sectional study of 8-year-old children to investigate the distribution and associations of AL, refractive error, and uncorrected visual acuity [11]. To date, few studies have examined the association between obesity-related indices and ocular parameters in children [12]. Because obesity is a modifiable factor, clarifying its relationship with ocular growth and refractive development may help identify potential targets for future public health research [13]. Accordingly, we aimed to examine the associations of ocular parameters with body composition parameters in children. Uncorrected visual acuity was included as a pragmatic functional outcome because it reflects the real-world visual consequences of uncorrected refractive status in school-age screening. Examining uncorrected visual acuity alongside SE and AL allowed us to evaluate whether body composition was associated not only with ocular biometry and refraction but also with unaided visual function.

## 2. Methods

This study was conducted among 8-year-old children enrolled in the JECS-Y. The enrollment period for participants in this study was from 7 July 2019 to 25 December 2022. The legal guardians of all participants provided written informed consent prior to study enrollment. The study was conducted in accordance with the Declaration of Helsinki. The JECS protocol was reviewed and approved by the Ministry of the Environment’s Institutional Review Board on Epidemiological Studies (IRB number: 100910001). The study was approved by the Institutional Review Board of the University of Yamanashi (No. 2070).

Ophthalmologic examinations included measurements of uncorrected visual acuity in both eyes. Uncorrected visual acuity was converted to the logarithm of the minimum angle of resolution (logMAR) units for statistical analysis. Noncycloplegic refraction was measured using an autorefractometer (NIDEK AR-F; NIDEK Co., Ltd., Gamagori, Japan). Refractive error was expressed as the spherical equivalent (SE), calculated as the spherical power plus half of the cylindrical power. The AL was measured in both eyes using an optical biometry device (NIDEK AL-SCAN; NIDEK Co., Ltd., Gamagori, Japan). Body composition was evaluated by measuring height, weight, muscle mass, and body fat percentage using a bioelectrical impedance analyzer (MC-780A; Tanita Corporation, Tokyo, Japan). The Rohrer index was calculated as body weight (kg)/height (cm^3^) × 10^7^. It is a physique index commonly used to assess obesity in Japanese school-aged children, with values of 115 to <145 generally considered to represent a normal physique. According to the conventional Japanese classification, Rohrer index values < 100 indicate underweight, 100 to <115 mild underweight, 115 to <145 normal physique, 145 to <160 overweight, and ≥160 obesity. Although body mass index (BMI) is widely recognized as an indicator of obesity in adults, it may not accurately reflect adiposity in children [14,15,16]. Therefore, alternative indices, including the Rohrer index, have been applied in pediatric populations [17].

### Statistical Analysis

To avoid inter-eye correlation, only right-eye data were included in the primary analyses. Similar results for the left eye are presented in the Supplementary Tables and Figures. Statistical analyses were conducted using EZR (R 4.6.1, Saitama Medical Center, Jichi Medical University, Saitama, Japan), a graphical user interface for R(R 4.6.1) Commander with customized statistical functions. Continuous variables are summarized as means ± standard deviations (SDs), whereas categorical variables are presented as frequencies and percentages. Baseline characteristics were compared between boys and girls using the Mann–Whitney U test. Pearson’s correlation coefficients were calculated to examine the associations of refractive error, AL, and uncorrected visual acuity with height, weight, muscle mass, body fat percentage, and the Rohrer index. Separate multiple linear regression models were constructed for refractive error, AL, and uncorrected visual acuity. The Rohrer index, muscle mass, and sex were included as independent variables in each model. Multicollinearity among the independent variables was assessed using variance inflation factors (VIFs). Unstandardized regression coefficients (β) with 95% confidence intervals (CIs) were reported. Because this was an exploratory analysis and the ocular and body composition variables were correlated, *p*-values were not adjusted for multiple testing; therefore, statistically significant findings, especially those with small effect sizes, were interpreted cautiously. Correlation coefficients with absolute values < 0.10 were considered negligible and were not interpreted as evidence of a meaningful linear relationship, irrespective of statistical significance. All statistical tests were two-sided, and *p*-values < 0.05 were considered statistically significant.

## 3. Results

### 3.1. Subject Demographics

Baseline characteristics of the study participants are shown in Table 1. A total of 2036 children were initially assessed, of whom 170 were excluded because AL measurements were unavailable. Consequently, 1866 children were included in the final analysis, comprising 921 boys and 945 girls. The number of participants by survey year was 233 in 2019, 550 in 2020, 696 in 2021, and 387 in 2022. The mean Rohrer index for the entire cohort was 128.3 ± 16.0. Overall, 1307 children (70.0%) were classified as within the normal range, 320 children (17.1%) as lean or underweight, and 239 children (12.7%) as overweight or obese. Sex-based comparisons revealed that height, body weight, muscle mass, and AL were significantly greater in boys, whereas body fat percentage was significantly higher in girls. The Rohrer index and SE did not differ significantly between boys and girls.

### 3.2. Correlation Between the Rohrer Index and SE, Visual Acuity logMAR, or AL

**Figure 1:**
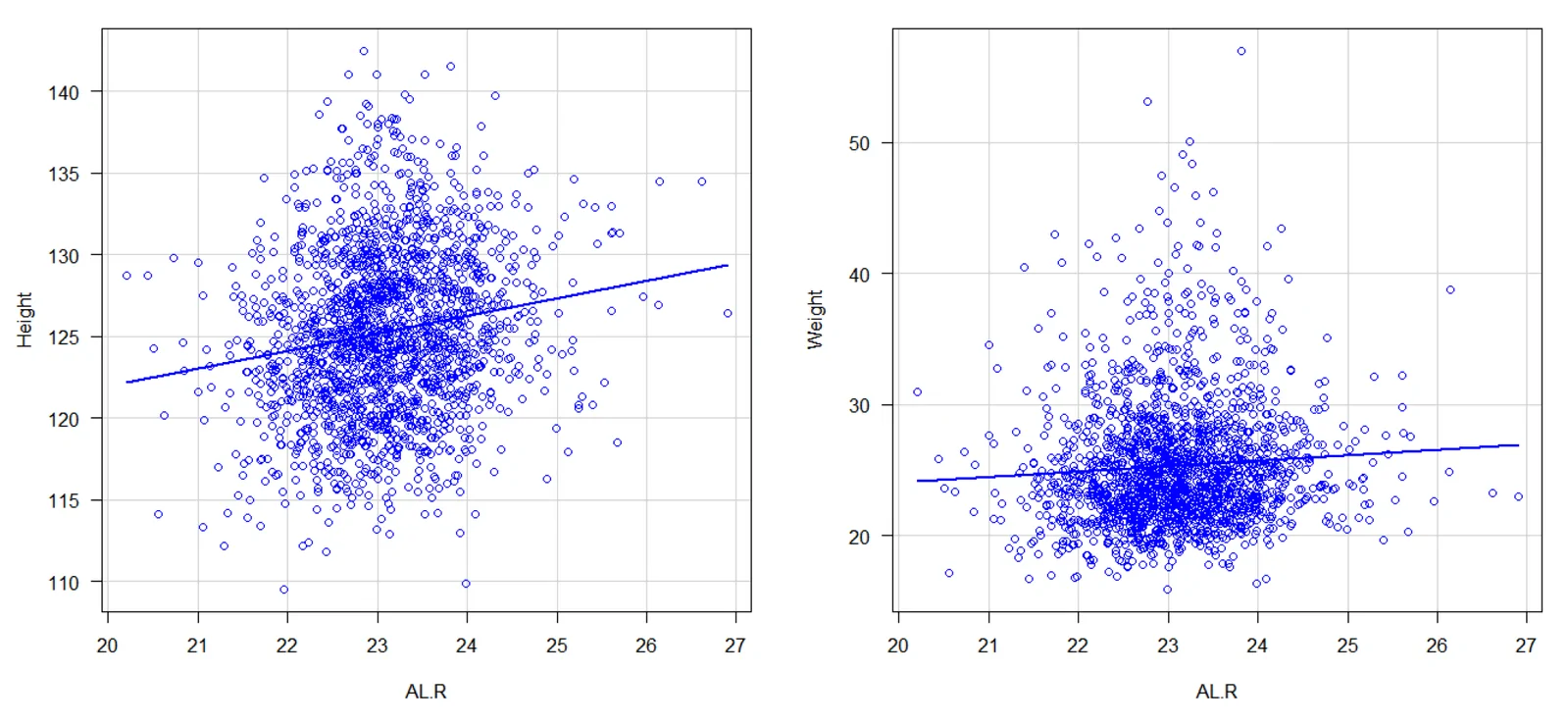
Relationships of AL, SE, and logMAR with the Rohrer index. Pearson’s correlation coefficients between the Rohrer index and AL, SE, and logMAR were close to zero. Although the unadjusted *p*-values were below 0.05, the magnitudes of the correlations were negligible.

The Pearson correlation coefficients between the Rohrer index and AL (r = −0.06), SE (r = 0.05), and uncorrected visual acuity expressed as logMAR (r = −0.07) were all close to zero, indicating no meaningful linear correlations. Although the corresponding unadjusted *p*-values were below 0.05, the magnitude of each correlation was negligible (Table 2; Figure 1).

### 3.3. Correlation Between AL and Anthropometric Parameters

**Figure 2:**
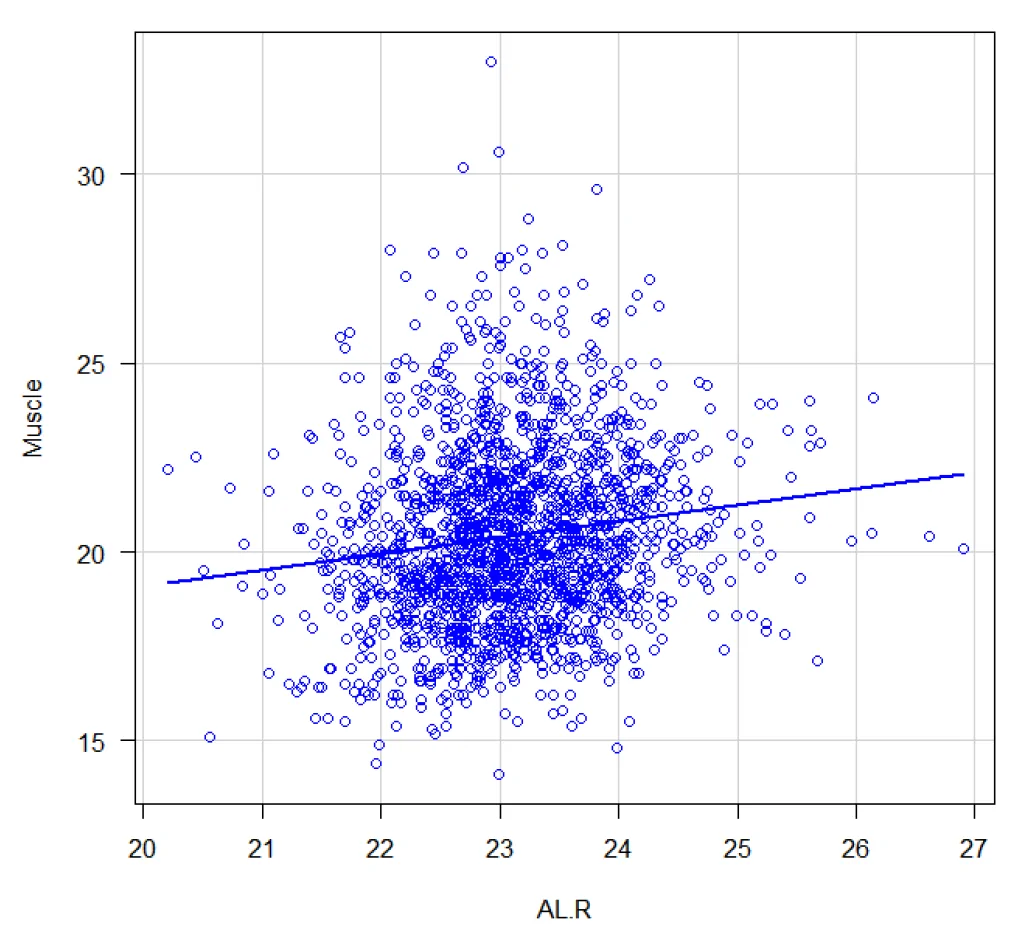
Relationships of AL with body composition parameters. AL showed small positive correlations with height and muscle mass, whereas the correlation with weight was negligible.

AL showed small positive correlations with height (r = 0.17, *p* < 0.001) and muscle mass (r = 0.14, *p* < 0.001). Although the correlation between AL and body weight had an unadjusted *p*-value below 0.05 (r = 0.07, *p* = 0.004), its magnitude was negligible. No meaningful correlations were observed between body fat percentage and AL, SE, or logMAR (Table 2; Figure 2).

### 3.4. Multiple Regression Analysis

Table 3 summarizes the results of the multiple linear regression analyses. After adjustment for muscle mass and sex, the regression coefficients for the Rohrer index were small for SE (β = 0.005 D per unit, 95% CI: 0.001 to 0.009, *p* = 0.021), AL (β = −0.006 mm per unit, 95% CI: −0.009 to −0.004, *p* < 0.001), and uncorrected visual acuity expressed as logMAR (β = −0.001 per unit, 95% CI: −0.002 to −0.0003, *p* = 0.006). Although the corresponding *p*-values were below 0.05, the estimated changes were small and of uncertain clinical relevance. Muscle mass and sex were independently associated with AL but not with SE or logMAR. Similar findings were observed for the left eye (Supplementary Tables). The explanatory power of the models was modest for AL (adjusted R^2^ = 0.125) and very limited for SE and logMAR (adjusted R^2^ = 0.001 and 0.004, respectively).

## 4. Discussion

### 4.1. Summary of Principal Findings

In this study, we examined the relationships of AL, SE, and logMAR with body composition parameters in 8-year-old children. The correlation coefficients between the Rohrer index and SE, logMAR, and AL were close to zero. Therefore, despite nominally significant *p*-values, the present data do not support meaningful linear correlations between the Rohrer index and these ocular parameters. AL showed small positive correlations with height and muscle mass, whereas the correlation with weight was negligible and body fat percentage was not meaningfully correlated with AL. In the multivariable analyses, muscle mass and sex remained associated with AL after adjustment for the Rohrer index, but the explanatory power of the models was limited. Similar results were observed for both eyes. Boys had greater height, weight, muscle mass, and AL than girls, whereas girls had a higher body fat percentage. Among the ocular outcomes, AL provides the most robust evidence because it is an objective biometric measure that is not affected by accommodation. The AL findings suggest that body composition has, at most, limited cross-sectional associations with ocular size in this cohort. However, because AL was measured at a single time point, these data do not directly assess ocular growth and should not be interpreted as proof that body composition has no effect on longitudinal axial elongation. In contrast, the SE results require particular caution because noncycloplegic refraction in children may be shifted in the myopic direction by accommodation. Uncorrected visual acuity was analyzed as a complementary functional outcome rather than as an independent measure of ocular growth; because it is largely determined by uncorrected refractive error, its findings should be interpreted together with the SE results.

### 4.2. Comparison with Previous Studies

Several previous studies have investigated the relationships between anthropometric or body composition parameters and ocular measurements, including AL and refractive error. Rahi et al. reported that greater height and lower BMI during late childhood and early adolescence were associated with earlier onset and greater severity of myopia, independent of sex, and that individuals with high myopia participated less frequently in sports [18]. Chen et al. examined adolescents aged 12–19 years and demonstrated significant associations of myopia with BMI, weight groups, and BMI categories; however, no significant association with height was observed, and the relationship between BMI and myopia was non-linear [19]. These findings differ from ours, in which AL showed more consistent associations with body composition than refractive error. Other studies have reported inconsistent associations between anthropometric characteristics and refractive error, with some demonstrating no association and others reporting modest relationships with body size or obesity [20,21,22]. Several factors may account for these discrepancies, including differences in participant age, ethnic background, and the indices used to assess obesity. Axial elongation is a characteristic feature of myopic eyes; however, during childhood, axial elongation also reflects normal physiological ocular growth. Therefore, physiological ocular growth should be distinguished from pathological axial elongation associated with myopia. Several longitudinal studies have demonstrated a significant association between height growth and AL growth during childhood and adolescence [23,24,25]. In contrast, evidence regarding the relationships between physiological somatic growth and AL, and between physical growth and refractive error, remains inconsistent across studies [20,21,22,26]. Accordingly, the relationship between height and refractive error may be more complex than that between height and physiological AL growth. It is well recognized that the growth velocities of both AL and height decrease with age. AL exhibits rapid growth around 8 years of age, whereas height reaches its peak growth velocity at approximately 10 years of age [27]. The younger age of the participants in the present study (8 years), compared with those in previous reports, may partly explain the differences observed. Consistent with this age-dependent effect, Bruce et al. reported that increases in height among adolescents aged 15 years were associated with decreases in SE and increases in AL, and that body weight was also associated with axial elongation [28]. Northstone et al. further reported that height growth trajectories over a 10-year period during adolescence were strongly positively associated with AL at 15 years of age [29]. The present analysis is cross-sectional; however, because the JECS is an ongoing prospective birth cohort, future follow-up of this cohort will enable investigation of longitudinal ocular growth, refractive development, and somatic growth.

### 4.3. Obesity-Related Indices and Ocular Parameters

In the present study, the correlation coefficients of the Rohrer index with AL, SE, and uncorrected visual acuity were all close to zero. Therefore, the present findings do not provide evidence of meaningful linear relationships between the Rohrer index and these ocular parameters, despite nominally significant *p*-values. The AL result is the most reliable of these findings because optical biometry is unaffected by accommodation. The small magnitude of the association between the Rohrer index and AL suggests that body composition has, at most, a limited cross-sectional relationship with ocular size at age 8. This conclusion concerns axial size at a single examination and not longitudinal ocular growth. Conversely, the SE findings are less definitive because noncycloplegic autorefraction may overestimate myopic refractive error in children. Previous studies have reported inconsistent findings regarding obesity-related indices and myopia [18,19,20,21,22]. Differences in participant age, ethnic background, definitions of obesity, cycloplegia, and study design may contribute to this inconsistency. The current cross-sectional results do not support mechanistic inferences linking the Rohrer index to refractive development. Longitudinal studies using validated age- and sex-specific measures of adiposity and cycloplegic refraction are needed to clarify whether obesity-related factors contribute to ocular growth or refractive development in children.

### 4.4. Factors Associated with Myopia Development

Children’s growth and development are influenced by complex interactions between genetic predisposition and environmental factors. Increased near-work activities related to education, reduced time spent outdoors, and decreased exposure to ambient light have been widely recognized as important contributors to myopia development. In addition, several prenatal and early-life factors—including advanced maternal age at childbirth, intrauterine growth restriction relative to gestational age, continued maternal smoking during pregnancy, changes in socioeconomic status, and reduced breastfeeding rates—have also been suggested to influence myopia development [30,31,32,33]. Given the rapidly increasing prevalence of myopia, a better understanding of the factors associated with ocular growth and refractive development may help inform future preventive strategies.

### 4.5. Sex Differences

In the present study, boys had significantly longer AL than girls, whereas the associations between obesity-related indices and ocular parameters were generally comparable between the sexes. This finding is consistent with our previous report [11]. Saw et al. also reported that, after adjustment for refractive error, age, school, height, parental myopia, and gestational age, mean head circumference was significantly larger in boys than in girls [34]. These findings suggest that certain aspects of ocular and somatic growth may differ between sexes. In the present analysis, SE did not differ significantly between boys and girls after adjustment for body composition parameters. This absence of a clear sex difference in refractive status is broadly consistent with Chen et al., who found only a small, statistically nonsignificant difference in myopia prevalence between adolescent girls (31.52%) and boys (28.37%) [19]. Nevertheless, differences in age and refraction methods limit direct comparison between the studies. Further studies are warranted to clarify potential sex-specific differences in ocular and refractive development.

### 4.6. Assessment of Obesity in Children

In the present study, the Rohrer index was selected because it is commonly used to evaluate body proportionality in Japanese school-aged children. Although body mass index (BMI) is widely used as an indicator of obesity in adults, adult BMI cutoff values are not appropriate for children because BMI varies considerably with age and growth [15,35,36]. The present results do not establish that the Rohrer index is superior to BMI or other measures of adiposity for evaluating ocular outcomes. Age- and sex-specific pediatric reference values and direct measures of body composition should be considered in future studies.

### 4.7. Limitations

Several limitations of this study should be acknowledged. First, this was a cross-sectional analysis, which precludes any inference regarding causal relationships between body composition parameters and ocular outcomes. Second, the study population consisted exclusively of Japanese children, limiting generalizability to populations with different body composition, growth patterns, and refractive characteristics. Third, refractive error was measured without cycloplegia; accommodation may therefore have resulted in an overestimation of myopia in some children. Fourth, multiple correlated ocular and body composition variables were examined without adjustment for multiple testing. Accordingly, *p*-values below 0.05 should be regarded as nominal, particularly when the corresponding effect sizes were negligible. Finally, the correlations of the Rohrer index with SE, logMAR, and AL were close to zero, and the regression models explained very little variance in SE and logMAR. These findings should not be interpreted as evidence of clinically meaningful relationships. Further longitudinal studies involving diverse populations, cycloplegic refraction, and validated pediatric measures of adiposity are warranted.

## 5. Conclusions

In this cross-sectional study of 8-year-old Japanese children, the Rohrer index showed no meaningful linear correlation with AL, SE, or uncorrected visual acuity despite nominally significant *p*-values. AL showed small positive correlations with height and muscle mass, whereas no meaningful relationship was observed with body fat percentage. Overall, the small effect sizes and limited explanatory power of the models preclude strong conclusions regarding the relationship between body composition and ocular growth. Longitudinal studies are needed to clarify the relationships among somatic growth, ocular growth, and refractive development during childhood.

## Figures and Tables

**Table 1:** Demographics of enrolled subjects.

Characteristic	Overall, N = 1866	Boys, N = 921	Girls, N = 945	*p*-Value
Rohrer index, mean ± SD	128.3 ± 16.0	128.7 ± 16.3	127.9 ± 15.7	0.27
<100	8 (0.4%)	3 (0.3%)	5 (0.5%)	
100–114	312 (16.7%)	114 (12.4%)	168 (17.8%)	
115–144	1307 (70.0%)	659 (71.6%)	648 (68.6%)	
145–159	153 (8.2%)	65 (7.1%)	88 (9.3%)	
≥160	86 (4.6%)	50 (5.4%)	36 (3.8%)	
Height, cm	125.3 ± 5.1	125.6 ± 5.1	124.9 ± 5.1	0.002
Weight, kg	25.4 ± 4.8	25.7 ± 5.0	25.1 ± 4.7	0.005
Muscle mass, kg	20.4 ± 2.4	20.9 ± 2.2	20.0 ± 2.4	<0.001
Body fat percentage, %	14.4 ± 6.6	13.6 ± 7.1	15.2 ± 5.9	<0.001
AL, mm	23.1 ± 0.8	23.4 ± 0.7	22.8 ± 0.8	<0.001
SE, D	−0.52 ± 1.28	−0.52 ± 1.19	−0.53 ± 1.36	0.89

AL and SE values represent measurements obtained from the right eye only. Continuous variables are presented as means ± standard deviations (SDs), and categorical variables as *n* (%). A two-sided *p*-value < 0.05 was considered statistically significant.

**Table 2:** Pearson correlation analysis of the Rohrer index, body composition parameters, refractive error, visual acuity, and axial length.

Outcome	Parameter	Correlation Coefficient (95% CI)	*p*-Value
AL	Rohrer index	−0.06 (−0.10 to −0.01)	0.02
AL	Muscle mass	0.14 (0.10 to 0.19)	<0.001
AL	Weight	0.07 (0.02 to 0.11)	0.004
AL	Height	0.17 (0.12 to 0.21)	<0.001
AL	Fat percentage	−0.04 (−0.08 to 0.01)	0.10
SE	Rohrer index	0.05 (0.01 to 0.10)	0.02
SE	Muscle mass	0.01 (−0.03 to 0.06)	0.53
SE	Weight	0.03 (−0.02 to 0.07)	0.26
SE	Height	−0.02 (−0.06 to 0.03)	0.49
SE	Fat percentage	0.03 (−0.01 to 0.08)	0.15
logMAR	Rohrer index	−0.07 (−0.11 to −0.02)	0.005
logMAR	Muscle mass	−0.02 (−0.07 to 0.02)	0.30
logMAR	Weight	−0.03 (−0.08 to 0.01)	0.19
logMAR	Height	0.02 (−0.02 to 0.07)	0.29
logMAR	Fat percentage	−0.03 (−0.08 to 0.01)	0.15

AL and SE values represent measurements obtained from the right eye only. Correlations were assessed using Pearson’s correlation coefficient.

**Table 3:** Multiple linear regression analysis of ocular parameters using the Rohrer index, muscle mass, and sex as explanatory variables.

Outcome	Predictor	β	95% CI	*p*-Value
AL	Rohrer index	−0.006	−0.009 to −0.004	<0.001
AL	Muscle mass	0.049	0.032 to 0.065	<0.001
AL	Sex	−0.476	−0.545 to −0.408	<0.001
SE	Rohrer index	0.005	0.001 to 0.009	0.021
SE	Muscle mass	−0.008	−0.036 to 0.021	0.59
SE	Sex	−0.016	−0.135 to 0.103	0.79
logMAR	Rohrer index	−0.001	−0.002 to −0.0003	0.006
logMAR	Muscle mass	0.002	−0.004 to 0.007	0.55
logMAR	Sex	0.017	−0.005 to 0.039	0.13

Regression coefficients are unstandardized β values. Multicollinearity was assessed using variance inflation factors (all VIFs < 2).

## Data Availability

The data are not publicly available because of ethical restrictions and the legal framework governing personal information protection in Japan. Public deposition of data containing personal information is prohibited under the Act on the Protection of Personal Information (Act No.57 of 30 May 2003, amendment on 9 September 2015). In addition, the Ethical Guidelines for Medical and Health Research Involving Human Subjects enforced by the Japan Ministry of Education, Culture, Sports, Science and Technology and the Ministry of Health, Labour and Welfare also restrict the open sharing of the epidemiologic data. All inquiries about access to data should be sent to rshinohara@yamanashi.ac.jp. The person responsible for handling enquiries sent to this e-mail address is Dr Ryoji Shinohara, director of Koshin Unit Center for JECS.

## Supplementary Materials

The following supporting information can be downloaded at https://www.mdpi.com/article/10.3390/jcm15176883/s1, Table S1: Single regression analysis of Rohrer index, body composition, refractive errors, and Axial Length of the left eye; Table S2: Multiple regression analysis with refractive error and Rohrer index, sex, and Body composition as explanatory variables of the left eye; Figure S1: The correlation of spherical equivalent with the Rohrer index in the left eye; Figure S2: The correlation of LogMAR with the Rohrer index in the left eye; Figure S3: The correlation of Axial Length with the Rohrer index in the left eye; Figure S4a: The correlation of Axial Length of the left eye with height; Figure S4b: The correlation of Axial Length with muscle mass in the left eye; Figure S4c: The correlation of Axial Length with weight.
