# Supplementary material for: Relationships of Body Composition with Refractive Error and Axial Length in 8-Year-Old Japanese Children
Source: J Clin Med. 2026 Sep 5;15(17):6883. doi: 10.3390/jcm15176883 (PMC13566156; doi:10.3390/jcm15176883)
Supplement: Supplementary file 1 [file jcm-15-06883-s001.zip › jcm-4487778-supplementary.pdf]

Supplementary Table S1: Single regression analysis of Rohrer index, body composition, refractive errors, and Axial Length of the left eye.

		Left eye	
		correlation coefficient (95%CI)	p-value
SE	Rohrer<br>index<br>Muscle mass<br>Weight<br>Height<br>Fat	0.06 (0.013-0.104)<br>0.02 (-0.026-0.065)<br>0.20 (-0.016-0.075)<br>-0.02 (-0.064-0.027)<br>0.03 (-0.014-0.077)	0.01*<br>0.40<br>0.20<br>0.42<br>0.18
logMAR	Rohrer<br>index<br>Muscle mass<br>Weight<br>Height<br>Fat	-0.07 (-0.116- -<br>0.026)<br>-0.03 (-0.076-0.015)<br>-0.04 (-0.083-0.007)<br>0.02 (-0.026-0.065)<br>-0.04 (-0.089-0.002)	0.002*<br>0.19<br>0.10<br>0.41<br>0.06
AL	Rohrer<br>index<br>Muscle mass<br>Weight<br>Height<br>Fat	-0.06 (-0.104- -<br>0.014)<br>0.14 (-0.094-0.184)<br>0.06 (0.014-0.105)<br>0.16 (0.117-0.206)<br>-0.04 (-0.090-0.001)	0.01*<br><0.001*<br>0.01*<br><0.001*<br>0.06

P-values were calculated using Pearson's product-moment correlation coefficient.
SE= spherical equivalent, AL= Axial Length

Supplementary Table S2 Multiple regression analysis with refractive error and Rohrer index, sex, and Body composition as explanatory variables of the left eye.

response variable	Rohrer		Muscle		Fat		Sex	
	β	p	β	p	β	p	β	p
SE-L	0.15	0.01*	0.008	0.78	-0.11	0.06	0.008	0.81
AL-L	-0.28	<0.001*	0.11	<0.001*	0.19	<0.001*	-0.33	<0.001*
logMAR-L	-0.15	0.002*	-0.007	0.81	0.09	0.06	0.01	0.53

The value of $p<0.05$ is the difference between the statistical and intentional values.

Supplementary Figure S1: The correlation of spherical equivalent with the Rohrer index in the left eye

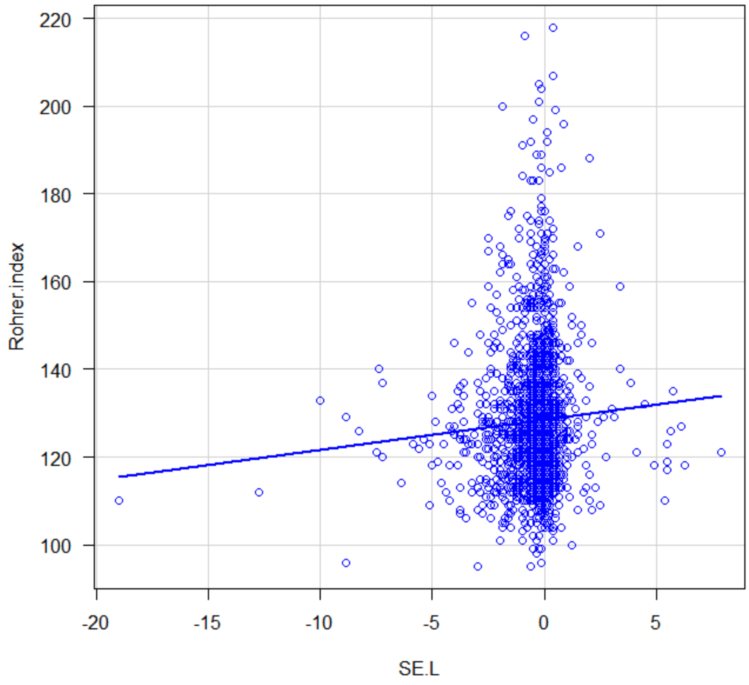

The Rohrer index showed a significant positive correlation with spherical equivalent (r = 0.06, p = 0.01, Pearson’s correlation coefficients)

Supplementary Figure S2: The correlation of LogMAR with the Rohrer index in the left eye

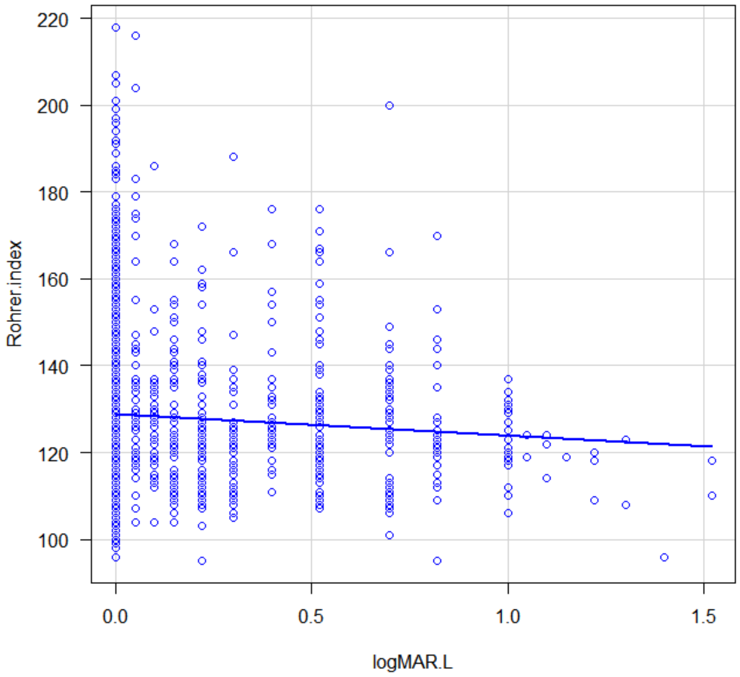

The Rohrer index was significantly negatively correlated with logMAR ($r = -0.07$, $p = 0.002$, Pearson's correlation coefficients).

Supplementary Figure S3: The correlation of Axial Length with the Rohrer index in the left eye

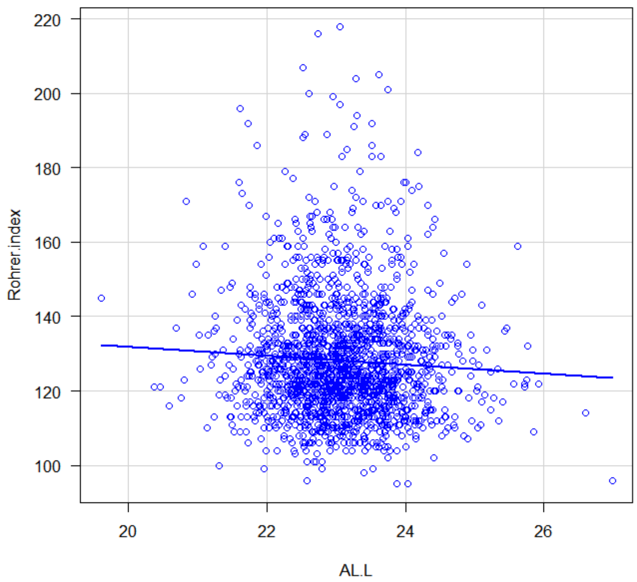

The Rohrer index was significantly negatively correlated with AL ($r = -0.06$, $p = 0.01$, Pearson's correlation coefficients).

Supplementary Figure S4a: The correlation of Axial Length of the left eye with height

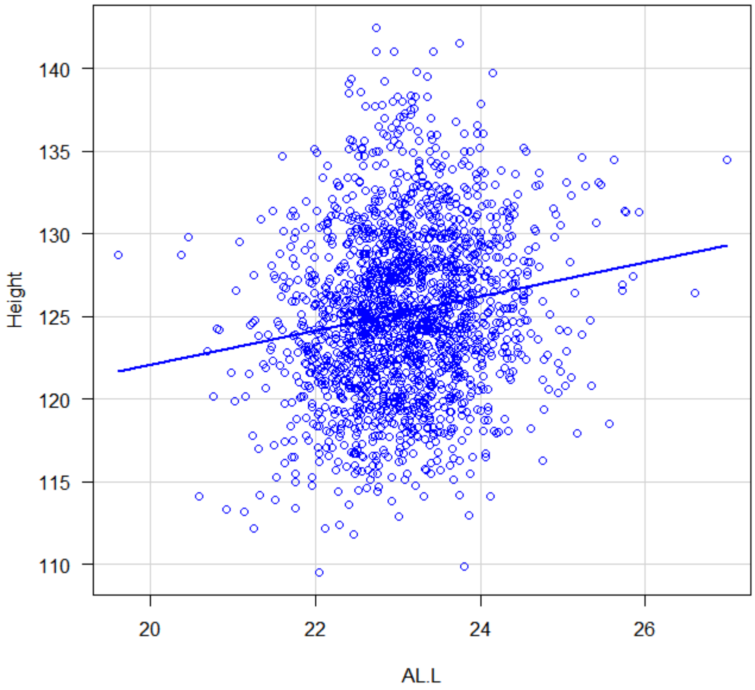

There is a significant positive correlation between axial length and height ($r = 0.16$, $p < 0.001$, Pearson's correlation coefficients).

Supplementary Figure S4b: The correlation of Axial Length with muscle mass in the left eye

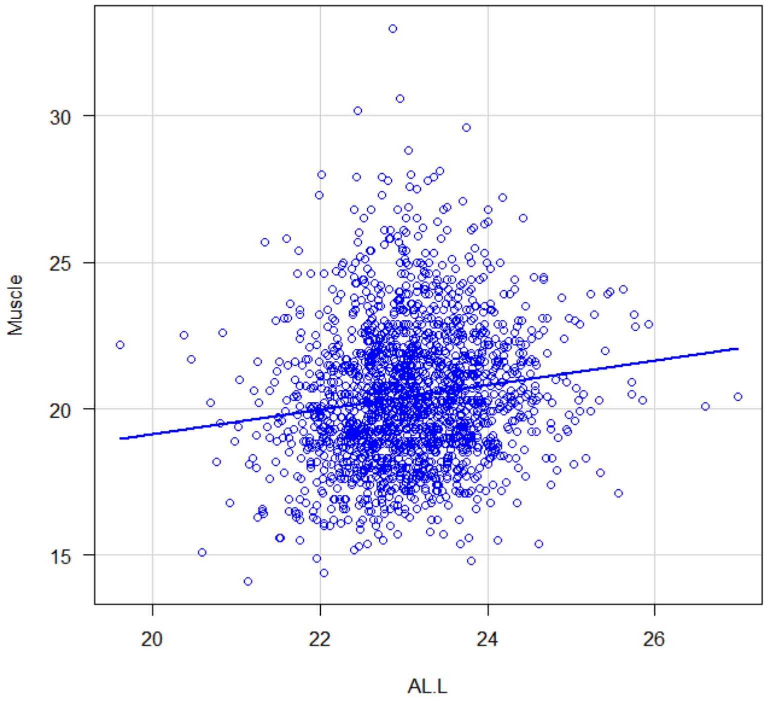

There is a significant positive correlation between axial length and and muscle mass (r = 0.14, p < 0.001, Pearson’s correlation coefficients).

Supplementary Figure S4c: The correlation of Axial Length with weight

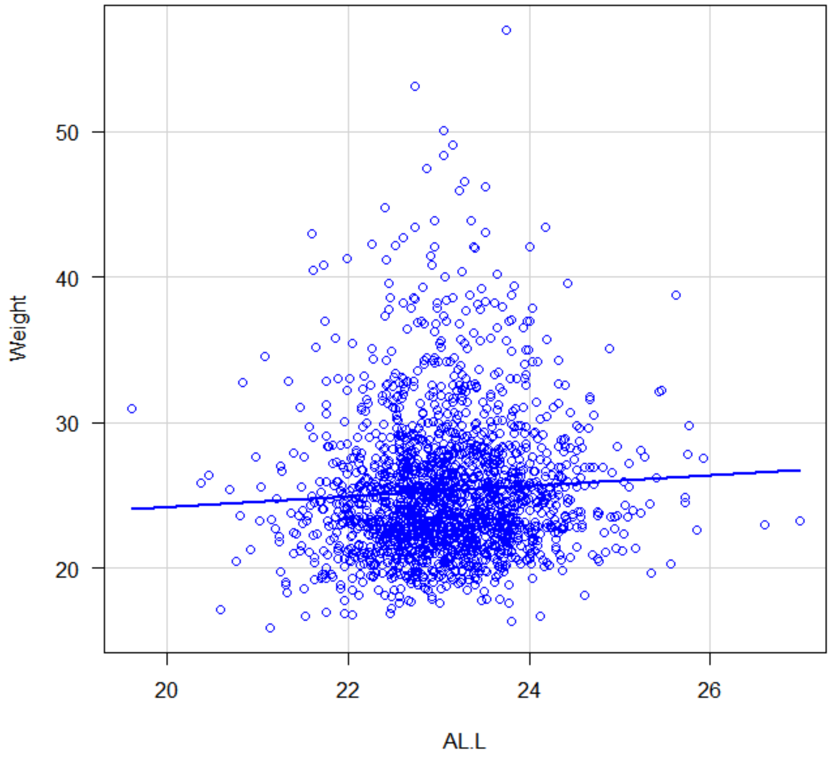

There is a significant positive correlation between axial length and muscle mass (left eye: r = 0.14, p < 0.001, Pearson's correlation coefficients).
